# Wafer‐Scale Growth and Transfer of High‐Quality MoS_2_ Array by Interface Design for High‐Stability Flexible Photosensitive Device

**DOI:** 10.1002/advs.202405050

**Published:** 2024-07-07

**Authors:** Bingchen Lü, Yang Chen, Xiaobao Ma, Zhiming Shi, Shanli Zhang, Yuping Jia, Yahui Li, Yuang Cheng, Ke Jiang, Wenwen Li, Wei Zhang, Yuanyuan Yue, Shaojuan Li, Xiaojuan Sun, Dabing Li

**Affiliations:** ^1^ Key Laboratory of Luminescence Science and Technology Chinese Academy of Sciences & State Key Laboratory of Luminescence and Applications Changchun Institute of Optics Fine Mechanics and Physics Chinese Academy of Sciences Changchun 130033 P. R. China; ^2^ Center of Materials Science and Optoelectronics Engineering University of Chinese Academy of Sciences Beijing 100049 P. R. China; ^3^ Key Laboratory of Automobile Materials MOE and School of Materials Science & Engineering and Electron Microscopy Center and International Center of Future Science and Jilin Provincial International Cooperation Key Laboratory of High‐Efficiency Clean Energy Materials Jilin University Changchun 130012 P. R. China; ^4^ School of Management Science and Information Engineering Jilin University of Finance and Economics Changchun 130117 P. R. China

**Keywords:** flexible device, graphene, interfacial adhesion, mechanical separation‐transfer, MoS_2_

## Abstract

Transition metal disulfide compounds (TMDCs) emerges as the promising candidate for new‐generation flexible (opto‐)electronic device fabrication. However, the harsh growth condition of TMDCs results in the necessity of using hard dielectric substrates, and thus the additional transfer process is essential but still challenging. Here, an efficient strategy for preparation and easy separation‐transfer of high‐uniform and quality‐enhanced MoS_2_ via the precursor pre‐annealing on the designed graphene inserting layer is demonstrated. Based on the novel strategy, it achieves the intact separation and transfer of a 2‐inch MoS_2_ array onto the flexible resin. It reveals that the graphene inserting layer not only enhances MoS_2_ quality but also decreases interfacial adhesion for easy separation‐transfer, which achieves a high yield of ≈99.83%. The theoretical calculations show that the chemical bonding formation at the growth interface has been eliminated by graphene. The separable graphene serves as a photocarrier transportation channel, making a largely enhanced responsivity up to 6.86 mA W^−1^, and the photodetector array also qualifies for imaging featured with high contrast. The flexible device exhibits high bending stability, which preserves almost 100% of initial performance after 5000 cycles. The proposed novel TMDCs growth and separation‐transfer strategy lightens their significance for advances in curved and wearable (opto‐)electronic applications.

## Graphical abstract

An interfacial adhesion regulation strategy is proposed for high‐quality growth and easy separation‐transfer of wafer‐scale MoS_2_ array onto the flexible substrate, which gains a high yield of ≈99.83%. The as‐fabricated flexible photodetector array exhibits high responsivity and imaging capability and also features excellent bending stability, which maintains almost 100% of its initial performance after 5000 cycles.

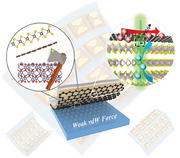

## 1 Introduction

2D materials, mainly graphene, transition metal disulfide compounds (TMDCs), and h‐BN, have attracted numerous attention for their ability in advanced (opto‐)electronic device manufacture.^[^ ^1^, ^2^, ^3^, ^4^ ^]^ Among them, the 2D TMDCs, such as MoS_2_, are regarded as the most promising candidates to replace conventional semiconductor systems and break Moore's law limitation because of their unique properties of atomic thickness, adjustable bandgap, and high carrier mobility.^[^ ^5^, ^6^, ^7^ ^]^ On the other part, the high in‐plane Young's modulus and ultrathin film of TMDCs trigger excellent mechanical strength and flexibility, and thus it is also qualified for flexible devices including curved and wearable applications, such as folding detectors and skin‐like sensors.^[^ ^8^, ^9^, ^10^ ^]^ For arbitrary flexible devices, the TMDCs supporting substrate is required to be soft materials and satisfy the bending needs. One direct and efficient strategy is TMDCs growth on flexible substrates like metal foil and organic polymer. However, the harsh TMDCs growth conditions, such as a sulfur‐rich atmosphere and high temperature, can induce serious chemical reactions or deformation of flexible substrates. Although Ahn. et al. recently reported the direct growth of MoS_2_ on ultrathin flexible parylene C substrate at a relatively low temperature (150 °C), the additional buffer layer of SiO_2_ dielectric material is still necessary.^[^ ^11^ ^]^ Meanwhile, the MoS_2_ layer exhibits a polycrystalline nature with a domain size within a few hundred nanometers. Up to now, the direct growth of high‐quality TMDCs on flexible substrates by widely adopted growth conditions still remains to be explored.

Hard dielectric materials with high chemical and thermal resistibility, typically for sapphire and quartz, are more commonly used as substrates for TMDCs growth. Due to their brittleness nature, further separation and transfer of TMDCs layer from the initial growth substrate onto a foreign flexible one is required for producing curved/wearable devices. The usual TMDCs transfer techniques could be divided into wet and dry routes. The wet transfer, involves supporting polymer coating and substrate chemical etching, which generally causes pollution by the residual polymer coating or etchant solution.^[^ ^12^, ^13^, ^14^ ^]^ The wet route fails in achieving timesaving and steady transfer of wafer‐scale TMDCs, since the substrate etching time is long enough and the mechanical strength of polymer coating might be insufficient. The dry transfer usually relies on a Polydimethylsiloxane (PDMS) stamp as the mediator, and thus the interfacial adhesion difference between PDMS/TMDCs and TMDCs/substrate is one of the crucial preconditions.^[^ ^15^, ^16^ ^]^ Meanwhile, the chemical vapor deposition (CVD) of TMDCs on dielectric substrates at high temperatures forms strong chemical bonding at the TMDCs/substrate interface, and the mechanical separation of an intact TMDCs layer by electrostatic adhesion of PDMS is challenging.^[^ ^17^ ^]^ For most situations, dry transfer introduces extra defects even film damage to the TMDCs.^[^ ^18^ ^]^ In order to solve these limitations, one effective strategy is to control the interfacial adhesion of TMDCs/substrate at a relatively low intensity, for example, weak van der Waals interaction. Apart from the surface/interface engineering of growth substrates with proper inserting layer, another together emerging challenge is the growth of high‐quality and uniform TMDCs on the modified substrate. However, to the best knowledge, the related strategy is still not well investigated, and thus it is necessary to design a novel interfacial configuration for high‐quality growth and easy transfer of TMDCs layer, which satisfies the fabrication of high‐performance flexible devices.

In this work, we demonstrated the controllable growth and easy mechanical separation‐transfer of the 2‐inch MoS_2_ layer by using graphene as the inserting layer, which leads to the weak van der Waals force at the growth interface. The pre‐deposition of MoO_x_ precursor and rapid annealing with SiO_2_ capping layer on the graphene‐modified sapphire substrate is proposed to optimize the crystalline quality and uniformity of MoS_2_ after the sulfurization process. In contrast to the bare sapphire substrate, the graphene inserting layer could maintain excellent integrity after the annealing and sulfurization process, triggering the MoS_2_ layer with higher flatness and absence of slit defects. The theoretical calculations reveal that the coupling effect between MoS_2_ and sapphire substrate is blocked by the graphene inserting layer, and the weak van der Waals interfacial adhesion results in a high separation‐transfer efficiency of patterned MoS_2_ array (≈99.83%). This separation‐transfer strategy also shows great applicability for the MoS_2_ grown by the reaction of sulfur and MoO_x_ powers, as well as other TMDCs, such as WS_2_. Meanwhile, the one‐step mechanical separation‐transfer technique lowers the difficulty of the flexible photodetector fabrication, and the separable graphene layer together with MoS_2_ serves as a photocarrier transportation channel, enhancing the device's responsivity by ≈10^5^. The mass production of MoS_2_ array enables the imaging functionality of flexible photodetectors, the letter “C” image shows high contrast due to the enhanced responsivity. This flexible MoS_2_‐based photodetector also exhibits outstanding bending stability, in which the photoresponse current almost maintains the starting value after 5000 bending cycles. This work provides a novel strategy for TMDCs growth and a path to flexible applications, which is meaningful for advances of new‐generation curved and wearable devices.

## 2 Results and Discussion

**Figure 1:**
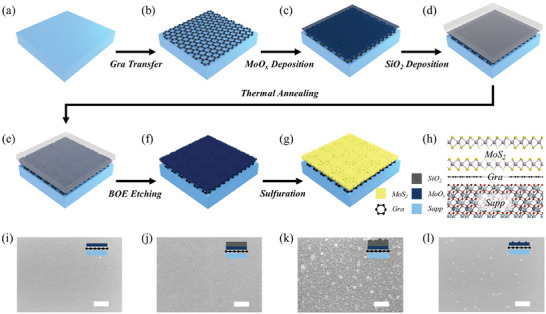
a‐g) Schematic diagrams of sapphire substrate modification and MoS_2_ growth, mainly including the graphene wet transfer, MoO_x_ precursor deposition, SiO_2_ capping layer deposition, high‐temperature annealing, chemical etching of SiO_2_ capping layer, and sulfuration of MoO_x_ precursor. h) Cross‐sectional configuration for multilayer MoS_2_ grown on graphene‐modified sapphire substrate in their atomic structures. i) SEM images of 5 nm MoO_x_ precursor deposited on graphene‐modified sapphire, j) after deposition of 50 nm SiO_2_ capping layer, k) after high‐temperature annealing at 800 °C for 30 min, l) after removing the capping layer by BOE solution. The scale bar is 500 nm.

The schematic diagrams for sapphire substrate modification and MoS_2_ growth are shown in **Figure** **1**a–g (see the Experimental Section for details). The CVD‐grown graphene on Cu foils is transferred onto the sapphire substrate by the wet transfer method, which acts as an inserting layer to promote the MoS_2_ properties and modify the interfacial adhesion (Figure 1a,b).^[^ ^19^ ^]^ After that, a MoO_x_ precursor layer with controlled thickness is deposited on the graphene inserting layer, followed by the deposition of a 50 nm SiO_2_ as the capping layer (Figure 1b–d). Then, the sample is annealed to enhance the crystalline quality of MoO_x_ precursor, and the SiO_2_ capping layer could ensure the stability of MoO_x_ film during the high‐temperature annealing (Figure 1d,e). As the annealing process finished, the SiO_2_ capping layer was removed by Buffered Oxide Etch (BOE) solution (Figure 1e,f). For the final step, the annealed MoO_x_ layer is sulfurized in a low‐pressure CVD system for the production of high‐quality and uniform MoS_2_ (Figure 1f,g). The schematic diagram for a cross‐sectional atomic configuration of as‐obtained multilayer MoS_2_ on a graphene‐modified sapphire substrate is shown in Figure 1 h.

The graphene inserting layer on the sapphire substrate offers a new platform for MoS_2_ growth, as mentioned above, it mainly includes the MoO_x_ layer deposition, high‐temperature annealing, and sulfurization processes. Since the capping layer plays a key role in the stable annealing of MoO_x_ precursor, the effects of capping layer thickness and material type are primarily evaluated by an optical microscope (OM) in Figure S1 (Supporting Information). For the SiO_2_ capping layer at 50  and 100 nm, the thicker one (Figure S1b, Supporting Information) appears numerous bubbles after the annealing. When the capping layer changes to 50 nm Si_3_N_4_ (Figure S1c, Supporting Information), it shows serious crack and self‐exfoliation. The possible reasons for these behaviors are discussed in the Supporting Information. Hence, we identify that the 50 nm SiO_2_ capping layer is the preferred parameter.

In order to confirm the surface variation during the whole MoS_2_ growth process, the scanning electron microscope (SEM) images of the sample after finishing each key step are shown in Figure 1i–l. For the deposition of 5 nm MoO_x_ on a graphene‐modified sapphire substrate (Figure 1i) and subsequent 50 nm SiO_2_ (Figure 1j), both of them have film flatness and continuity. It demonstrates that the as‐transferred graphene inserting layer is absent of residual pollutions and the MoO_x_ deposited by thermal evaporation has no trend to aggregate into clusters. On the other hand, the dense SiO_2_ capping layer could provide effective protection to the underneath MoO_x_ layer.^[^ ^20^ ^]^ After high‐temperature annealing at 800 °C (Figure 1k), the SiO_2_ capping layer becomes slightly coarsening and appears particle‐like distribution. The atomic force microscope (AFM) is applied to further investigate the annealed SiO_2_ capping layer, as shown in Figure S2 (Supporting Information). The height profile shows that the maximum particle height (≈16 nm) is lower than the thickness of the as‐deposited SiO_2_ capping layer. Therefore, the SiO_2_ particles only form at the surface but they still remain continuous below these particles, the schematic diagram of cross‐sectional structures is shown in the inset of Figure 1k. The SiO_2_ continuity is further confirmed by the excellent uniformity of MoO_x_ morphology after high‐temperature annealing (Figure 1l), attributing to the effective protection of SiO_2_ capping layer. As a comparison, an annealed MoO_x_ without SiO_2_ capping layer has been deformed into discrete clusters as shown in Figure S3 (Supporting Information).

**Figure 2:**
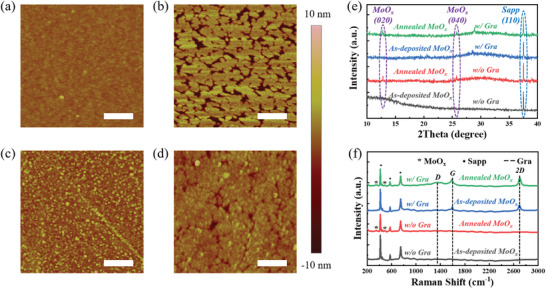
a) AFM images of the MoO_x_ films before and b) after annealing on bare sapphire, c) and d) are that on the graphene modified sapphire substrate. The scale bar is 200 nm. e) XRD 2θ scanning of these 4 samples in (a‐d), and the typical crystal planes of MoO_x_ and sapphire are marked. f) Raman spectra of the MoO_x_ precursor, showing the typical Raman bands of MoO_x_, graphene inserting layer, and sapphire substrate for the annealed MoO_x_ on graphene‐modified sapphire substrate.

The as‐deposited MoO_x_ precursor on bare sapphire substrate and that with graphene inserting layer is compared by AFM in **Figure** **2**a,c. The MoO_x_ on the bare sapphire exhibits a flatter morphology than that on the graphene, which is attributed to the different growth modes of MoO_x_ on these two surfaces because of the large surface energy difference.^[^ ^21^, ^22^, ^23^, ^24^ ^]^ Here, the MoO_x_ deposited on graphene tends to be an island‐liked Volmer‐Weber growth mode, in which the interaction among MoO_x_ molecules is stronger than that with graphene, resulting in many particles (Figure 2c).^[^ ^25^, ^26^ ^]^ As shown in Figure 2b, the MoO_x_ on bare sapphire changes into many patches with high‐density slits after the annealing with SiO_2_ capping layer. Considering about the original MoO_x_ thickness of 5 nm, the deep slits (≈10 nm) must lack MoO_x_ filling, and thus the annealed MoO_x_ on a bare sapphire substrate is fragmentized in film integrity. As for the MoO_x_ annealed on the graphene inserting layer (Figure 2d), only a few pinholes appear on the surface, and nearly the whole film maintains the continuity. According to the surface morphology variation, the compactness of MoO_x_ has been enhanced after the annealing, in which the energy‐driven molecule migration and rearrangement would contribute to the higher crystalline property of MoO_x_ precursor, further optimizing the MoS_2_ quality after the sulfuration process.^[^ ^27^, ^28^, ^29^ ^]^

The crystalline quality of MoO_x_ is evaluated by X‐ray diffraction (XRD) in Figure 2e. The XRD 2θ scanning shows typical MoO_x_ diffraction peaks at ≈12.8° and ≈25.7° for that after the annealing (both for that on bare sapphire and graphene inserting layer), corresponding to the crystal plane of (020) and (040).^[^ ^30^ ^]^ Due to the thin quantity (≈5 nm) of MoO_x_, the diffraction peak intensity is weaker than that of sapphire substrate. Similar results are obtained by Raman spectra in Figure 2f, only for the annealed MoO_x_, their typical bands could be detected (marked by an asterisk), which derives from the reported MoO_3_ and MoO_3‐x_.^[^ ^31^, ^32^, ^33^ ^]^ The enlarged Raman spectra of MoO_x_ on graphene/sapphire clearly exhibit the appearance of MoO_x_‐related bands after the annealing, as shown in Figure S4 (Supporting Information). Hence, the crystalline quality of MoO_x_ precursor is assuredly enhanced by the annealing treatment. Besides, in Figure S5 (Supporting Information), we design an experiment to demonstrate the excellent chemical stability of annealed MoO_x_ film. Here, two samples with 5 nm MoO_x_ are deposited on a graphene‐modified sapphire substrate, and the only difference is whether the MoO_x_ is annealed. After the samples are disposed of by BOE, the annealed MoO_x_ shows dark black color, but the unannealed sample is semitransparent (Figure S5a, Supporting Information). Meanwhile, the Raman spectra of the unannealed sample after sulfuration appeared no MoS_2_‐related bands (Figure S5b, Supporting Information), indicating that the as‐deposited MoO_x_ is resistless to the BOE solution and destroyed from the graphene inserting layer by this treatment. As for the annealed MoO_x_, the excellent stability results in the successful growth of MoS_2_.

The Raman spectra of graphene are also measured in Figure 2f, showing the typical G band and 2D band, as marked by the dashed lines. Since the intensity of G and 2D bands shows no obvious degradation after high‐temperature annealing, the main structure integrity of graphene could be ensured. On the other hand, the intensity of the defect‐related D band slightly increases, which might be induced by the few C atom vacancies in the graphene lattice during the high‐temperature annealing.^[^ ^34^ ^]^ These sites can improve the adhesion properties of MoO_x_ on the graphene‐inserting layer, changing the MoO_x_ growth into film‐preferred Frank‐Van der Merwe mode.^[^ ^24^, ^35^ ^]^ Therefore, the MoO_x_ particle on the graphene inserting layer has been obviously suppressed after the annealing treatment, as shown in Figure 2c,d.

**Figure 3:**
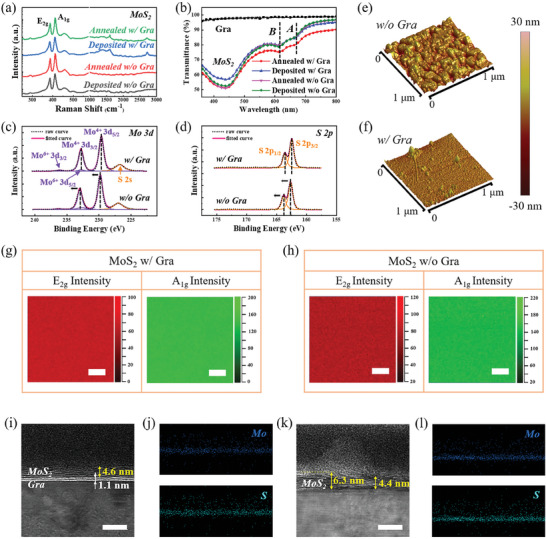
a) Raman spectra of four MoS_2_ samples, which are obtained by the sulfurization of MoO_x_ shown in Figure 2(a–d), the measured wavenumber of 200–3000 cm^−1^ covers the typical bands of MoS_2_, graphene inserting layer and sapphire substrate. b) Transmittance spectra of the MoS_2_ in (a), the A and B excitonic absorption of MoS_2_ is marked, and a graphene layer on sapphire serves as the reference. c) XPS core level spectra with deconvolution of Mo 3d and d) S 2p for MoS_2_ sulfurized from annealed MoO_x_ with and without graphene insertion. e) 3D AFM images of MoS_2_ without and f) with graphene inserting layer. g,h) Raman mapping of MoS_2_ E_2g_ and A_1g_ bands intensity, (g) and (h) is that sulfurized from annealed MoO_x_ with and without a graphene inserting layer. The scale bar is 2 µm. i,j) Cross‐sectional HRTEM images and EDX mapping of MoS_2_ grown with and k, l) without graphene inserting layer, clearly showing the layered MoS_2_ and graphene. The scale bar is 10 nm.

To obtain MoS_2_ materials, the MoO_x_ precursors are reacted with sulfur powder in a low‐pressure CVD system at 800 °C, which is identified as a sulfurization process. There are four controlled MoS_2_ samples according to the MoO_x_ presented in Figure 2a–d. As shown in **Figure** **3**a, with the annealing of MoO_x_ precursor, the intensity of typical MoS_2_ bands (E_2g_ and A_1g_) is slightly higher than that without annealing. The Raman measurement indicates the enhanced crystalline quality of MoS_2_, which benefits from the advantages created by MoO_x_ precursor annealing.^[^ ^36^ ^]^ The difference between E_2g_ and A_1g_ band position in Raman spectra reveals the thickness of MoS_2_. As shown in Figure S6 (Supporting Information), the MoS_2_ with a series of thicknesses is obtained by simply controlling the as‐deposited MoO_x_ precursor (from 2 to 7 nm). Hence, the proposed MoS_2_ growth strategy satisfies the high elasticity for the easy control of TMDCs properties. The transmittance spectra of MoS_2_ are shown in Figure 3b, and a graphene inserting layer on sapphire serves as the reference. The graphene layer exhibits a high transmittance through the entire wavelength, it reaches ≈97% at 550 nm, corresponding to the absorption value (2.3%) of a graphene layer.^[^ ^37^ ^]^ All of the MoS_2_ samples exhibit typical A and B exciton absorption peaks, among them, the MoS_2_ grown on graphene inserting layer from the annealed MoO_x_ precursor has the highest light absorption, which is favorable for its severing as the photosensitive absorber to fabricate a photodetector.

The X‐ray photoelectron spectroscopy (XPS) is used to investigate the chemical component of MoS_2_, the XPS survey scan is shown in Figure S7 (Supporting Information), and the core level spectrum of Mo 3d and S 2p are shown in Figure 3c,d, respectively. Since the annealing of MoO_x_ precursor is proved worthy for the high‐quality and uniform MoS_2_ growth, to evaluate the function of the graphene inserting layer, we just offer and compare two MoS_2_ samples of that annealed with and without graphene in the following sections. For the Mo 3d peak, it consists of Mo^4+^ 3d_5/2_, Mo^4+^ 3d_3/2_, Mo^6+^ 3d_5/2_, and Mo^6+^ 3d_3/2_, which are located at 229.63, 232.82, 232.77, and 236.14 eV for MoS_2_ grown with the graphene inserting layer. The Mo^6+^ peaks come from the Mo‐O bonding of MoO_x_, which is extremely weak in contrast to the Mo^4+^ peak intensity, proving that the sulfuration of MoO_x_ precursor is sufficient. As for that without graphene, the Mo^4+^ 3d_5/2_ and Mo^4+^ 3d_3/2_ shift to 229.84 and 233.01 eV. The S 2p peak could be deconvoluted into S 2p_3/2_ and S 2p_1/2_, which are located at 162.46 and 163.64 eV for MoS_2_ grown on graphene. Similarly to the Mo^4+^ peaks, they show the shift to 162.66 and 163.82 eV for that without graphene. Considering that the binding energy is sensitive to the material stress state, the shift of peaks in XPS spectra exhibits that the graphene inserting layer triggers weak van der Waals interaction between the MoS_2_ and sapphire substrate, so the direct formation of chemical bonding is suppressed. Although there is a thermal expansion coefficient difference, the stress in MoS_2_ could be efficiently released via the graphene inserting layer during the cooling procedure, existing as a stress‐free state in contrast to that grown without graphene.^[^ ^11^ ^]^ The interfacial field screening effect achieved by the graphene inserting layer will be confirmed by theoretical calculations in the following part. The ratio of Mo to S atom number is calculated by the XPS integral area of Mo 3d and S 2p peaks, and the value of ≈0.54 is approximately equal to the intrinsic ratio of MoS_2_ material.

The continuity and uniformity of MoS_2_ are evaluated by AFM and Raman mapping tests. As shown in Figure 3e,f, the AFM images indicate that the MoS_2_ surface morphology inherits the annealed MoO_x_ precursor without and with graphene (Figure 2b,d). Numerous slits appear for the MoS_2_ grown on a bare sapphire substrate, resulting in a high surface root‐mean‐square roughness of 3.76 nm. The results of Raman mapping in Figure 3g,h exhibit the similar surface properties of these two MoS_2_ films as that from AFM images, the distribution of typical E_2g_ and A_1g_ band intensity for that with a graphene inserting layer is more uniform and continuous. Therefore, it could be concluded that the graphene‐inserting layer plays a key role in optimizing the continuity and uniformity of MoS_2_ in the present growth strategy.

The lattice structures of MoS_2_ grown by the sulfurization of annealed MoO_x_ are confirmed by high‐resolution transmission electron microscopy (HRTEM). As shown in Figure 3i, the cross‐sectional HRTEM image of MoS_2_ with the graphene inserting layer exhibits clear layered structures, corresponding to ≈6 layers of MoS_2_, which is consistent with the Raman spectra in Figure 3a. The graphene‐inserting layer maintains the continuous layered structure, proving its survival ability after the high‐temperature MoO_x_ annealing and sulfurization process. In Figure 3k, the thickness of MoS_2_ without the graphene layer exhibits a large difference from 4.4  to 6.3 nm, which is induced by the numerous slits on MoS_2_, as shown in Figure 3e. More importantly, the growth interface between MoS_2_ and the sapphire substrate is not as clear as that grown with the graphene inserting layer, which might be evidence of strong interfacial coupling related to the direct interaction of MoS_2_ and sapphire. As shown in Figure 3j,l and Figure S8 (Supporting Information), the energy dispersive X‐ray spectroscopy (EDX) mapping of MoS_2_ shows the uniformity of Mo and S element distribution on the graphene inserting layer and bare sapphire, respectively. The zero atomic ratio of Si element on these two samples indicates that the SiO_2_ capping layer could be completely removed by BOE etching (Figure S9, Supporting Information), also revealed by the clean top surface in their HRTEM images (Figure 3i,k). As a result, this growth strategy for MoS_2_ is immune from Si‐related pollution. Meanwhile, the MoS_2_ with the graphene inserting layer was scraped and made into a solution, further dripping onto the copper mesh for the surface HRTEM test, as shown in Figure S10 (Supporting Information). The HRTEM image shows an apparent crystal lattice in these MoS_2_ crystal domains. However, since this MoS_2_ was prepared by the dropping method from the solution after ultrasonic dispersion, the domain size and orientation of MoS_2_ are different (Figure S10a, Supporting Information). The selected area electron diffraction (SAED) patterns show a relatively complex spot distribution (Figure S10b, Supporting Information), which still tends to arrange in the hexagonal symmetry, according with the crystal structure of MoS_2_. The MoS_2_ grown by the vapor reaction of sulfur and MoO_x_ powders on the sapphire substrate was also evaluated by the HRTEM and SAED measurements. The typical lattice structure is observed for MoS_2_ (Figure S11a, Supporting Information), and it exhibits clearer spot patterns in hexagonal symmetry (Figure S11b, Supporting Information), revealing the higher crystalline quality.

**Figure 4:**
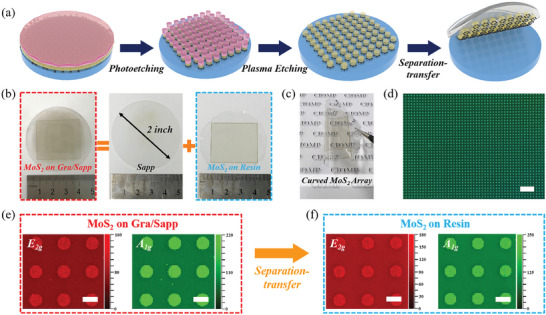
a) Schematic diagrams for the fabrication and separation‐transfer of wafer‐scale patterned MoS_2_ array. b) Photograph of a 2‐inch patterned MoS_2_ array before (on Gra/Sapp) and after separation‐transfer (on resin). c) Photograph of curved MoS_2_ by folding it on the opposite sides. d) OM image of the patterned MoS_2_ array on the resin. The scale bar is 200 µm. e) Raman mapping for E_2g_ and A_1g_ intensity of patterned MoS_2_ array on Gra/Sapp and f) on resin after the separation‐transfer. The scale bar is 20 µm.

By the growth interface design, we hope that the graphene inserting layer provides the weak van der Waals interaction between MoS_2_ and sapphire substrate, which makes it possible for separation and transfer of large‐area MoS_2_ by simply applying a mechanical force.^[^ ^38^ ^]^ For the convenience of calculating the separation‐transfer efficiency of MoS_2_, the film was processed into patterned array by the photoetching and plasma etching processes, as shown in **Figure** **4**a. After that, the ultraviolet (UV) photosensitive resin is spin‐coated on the patterned MoS_2_ array. The cured resin after being exposed to a UV lamp shows excellent flexibility, which could act as both a supporting layer for MoS_2_ separation and a flexible substrate for the following device fabrication. As shown in Figure S12 (Supporting Information), a contrast experiment is first carried out to evaluate the effect of the graphene inserting layer on weakening the interfacial adhesion. The result shows that only the MoS_2_ grown on the graphene inserting layer could be intactly separated, and the other part of MoS_2_ still remains on a bare sapphire substrate. As mentioned above, when the MoS_2_ directly contacts with sapphire substrate, the strong interfacial adhesion induced by the chemical bonding effect frustrates the mechanical separation. To further verify the effectiveness of the graphene inserting layer, the MoS_2_ grown by the vapor reaction of sulfur and MoO_x_ powders on the sapphire substrate with a half graphene layer coverage was also prepared and separated by using the UV resin, as shown in Figure S13 (Supporting Information). The MoS_2_ on the bare sapphire is difficult to be intactly separated, while the MoS_2_ grown on the other part with covered graphene shows successful separation, which reveals the applicability of this separation‐transfer strategy.

As shown in Figure 4b, the 2‐inch patterned MoS_2_ array is successfully separated and transferred onto flexible resin by the proposed separation‐transfer strategy in Figure 4a. It shows excellent flexibility, which can be easily bent and stuck on opposite sides (Figure 4c). For the wafer‐scale MoS_2_ array, it has a large amount of circular patterning units, recognized as ≈0.63 million (838 × 750). The OM image of the patterned MoS_2_ array transferred onto the resin is shown in Figure 4d, none defective unit is observed in the measured region. The SEM images of a single MoS_2_ unit before and after transfer are compared in Figure S14 (Supporting Information), the clear edge as well as intact geometry of the patterned MoS_2_ circle demonstrates the high separation‐transfer reliability. As shown in Figure 4e,f, the Raman mapping comparison between patterned MoS_2_ array on initial graphene‐modified sapphire substrate and later flexible resin further confirms the reliability of graphene inserting layer for the damage‐free separation‐transfer of MoS_2_. The typical Raman bands of E_2g_ and A_1g_ have consistent intensity and similar shape distribution for the patterned MoS_2_ circles on the flexible resin. In order to precisely obtain the separation‐transfer efficiency of the patterned MoS_2_ array, six different positions on the 2‐inch MoS_2_ wafer are measured in Figure S15 (Supporting Information) and the separation‐transfer efficiency is summarized in Table S1 (Supporting Information), which achieves a high yield of ≈99.83%. The separation‐transfer efficiency of this strategy on the MoS_2_ grown by the vapor reaction of sulfur and MoO_x_ powders and WS_2_ grown by sulfurization of WO_x_ film on the graphene inserting layer are verified, they were etched into a circle array before the UV resin coating. The corresponding Raman spectra in Figure S16a (Supporting Information) exhibit the typical Raman bands of TMDCs (MoS_2_ and WS_2_) and graphene inserting layer. The thickness of the patterned TMDCs array was measured by AFM (Figure S16b, Supporting Information). As shown in Figure S17 (Supporting Information), the separation‐transfer of the MoS_2_ and WS_2_ also achieves a high yield of over 99.38%. These results demonstrate the promising applicability of this separation‐transfer strategy for other 2D materials beyond the TMDCs. Furthermore, the low separation‐transfer failure of patterned TMDCs array ensures the mass production of high‐performance flexible (opto‐)electronic devices. In comparison with previously reported methods, the separation‐transfer strategy proposed in this work shows great advantages in device fabrication efficiency, yield, and size, as exhibited by the comparison in **Table** **1**.

**Table 1:** Comparison of previously reported MoS_2_ transfer methods with this work.

Target Substrate	Transfer Method	Features	Duration	for Array	Surface Quality/Yield	Size	Ref.
SiO_2_/Si	PMMA‐assisted wet transfer	Substrate etching and PMMA removing	96 h	No	Wrinkled/None	≈10 µm	[39]
Mica	PDMS‐assisted quasi‐dry transfer	Water soaking and PDMS heating	/	No	Damaged/None	1 cm	[40]
PET	PMMA‐assisted wet transfer	Substrate etching and PMMA removing	a few h	No	Good/None	<30 µm	[41]
PET	PMMA‐assisted wet transfer	Substrate etching and PMMA removing	/	Yes	Good/None	4 inches	[42]
Arbitrary materials	UV‐release tape and patterned template‐assisted mechanical dry transfer	Total dry process with additional patterned template	>4 h	Yes	Damaged/>80%	0.9 cm	[43]
Norland 63	Mechanical dry transfer by growth interface design	Simple transfer and total dry process	<5 s	Yes	Good/≈99.83%	2 inches	This work

**Figure 5:**
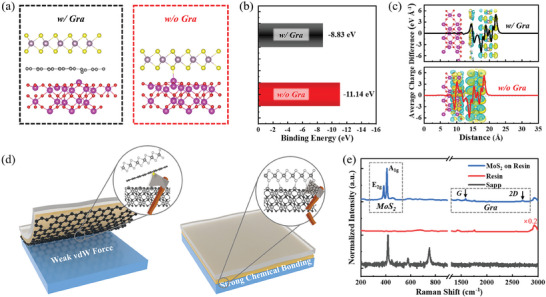
a) Cross‐sectional configuration of the calculated MoS_2_ interfacial structure models with and without the graphene inserting layer. b) Binding energy comparison and c) differential charge density distribution difference, in which the charge density distribution variation curves along the growth direction are plotted. d) Schematic diagrams for the easy separation‐transfer of MoS_2_ with the graphene inserting layer (weak van der Waals force), while it failed for the separation of that without graphene (strong chemical bonding). e) Raman spectra of MoS_2_ on resin after the separation‐transfer, both showing the bands related to MoS_2_ and graphene. The bare resin and sapphire substrate after MoS_2_ separation are measured as the reference.

The interfacial adhesion control mechanism of MoS_2_ growth by designing it with a graphene inserting layer is investigated by the first‐principles calculations. Since the defect‐related Raman D band of the graphene inserting layer has appeared after the MoS_2_ growth (Figure 3a), the graphene with a point defect of C vacancy is under consideration in the theoretical calculation. As shown in **Figure** **5**a, after the structure optimization, the final calculated MoS_2_ interfacial structure models of MoS_2_/graphene/sapphire and MoS_2_/sapphire are established. The inserting graphene layer triggers the spacial separation of MoS_2_ and sapphire substrate, which enhances their interaction distance. Although the graphene possesses a C vacancy, it has no chemical bonding with either top MoS_2_ or bottom sapphire substrate, demonstrating the weak van der Waals interaction. As for the MoS_2_/sapphire structure, an S atom of MoS_2_ has formed a strong chemical bonding with one of Al atoms in sapphire, which is directly related to the difficulty of MoS_2_ separation‐transfer.^[^ ^17^ ^]^ The binding energy of these two structures is calculated in Figure 5b, it is recognized that the lower binding energy corresponds to better structure stability, making it difficult for the structure change, typically for the MoS_2_ separation. The calculated binding energy of MoS_2_/graphene/sapphire is −8.83 eV, which is higher than −11.14 eV of MoS_2_/sapphire, showing that weak van der Waals force provided by graphene inserting layer results in the easy separation MoS_2_ from the sapphire substrate.

In order to further evaluate the coupling effect at the MoS_2_ growth interface, the differential charge density distribution is calculated, and the variation curve along the growth direction is plotted, as shown in Figure 5c. For the growth interface with the graphene inserting layer, the high‐density charge distribution only appears among the graphene and MoS_2_ layers, while no coupling effect is observed between the graphene and sapphire substrate. These results reveal that the coupling effect at MoS_2_/graphene interface is stronger than that of graphene/sapphire. Here, the absence of charge density at graphene/sapphire might be attributed to the C vacancy defect in graphene, which has screened the lattice field effect from the sapphire substrate. For the MoS_2_/sapphire structure, a high charge density distributes around MoS_2_ and sapphire substrate, which is induced by the direct chemical bonding formation between MoS_2_ and sapphire (see Figure 5a), and it is the main reason for the MoS_2_ separation failure (Figures S12 and S13, Supporting Information). According to the theoretical calculations, the vivid schematic diagrams for MoS_2_ separation‐transfer models with and without a graphene inserting layer are proposed, as shown in Figure 5d. Thanks to the weak van der Waals force, the easy separation‐transfer obtains a large‐area intact MoS_2_ by designing the growth interface with a graphene inserting layer. However, the adhesiveness generated by resin is not sufficient to break the strong chemical bonding between MoS_2_ and sapphire. Since the coupling effect of MoS_2_/graphene is stronger than that of graphene/sapphire, thus, the graphene theoretically prefers to be together separated with MoS_2_. Experimentally, as shown in Figure 5e, the Raman spectra of MoS_2_ on resin after separation‐transfer actually exhibit both typical bands of MoS_2_ and graphene (blue line). On the other hand, after the MoS_2_ separation‐transfer, the sapphire substrate is absent of these two materials (black line).

**Figure 6:**
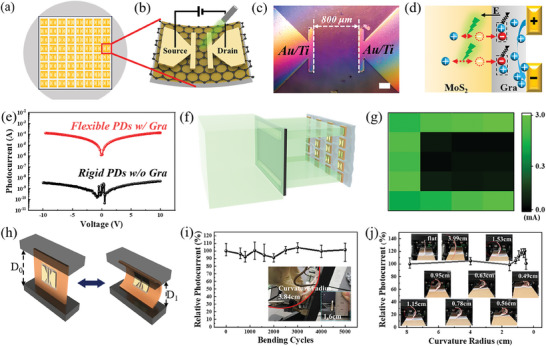
a) Schematic diagrams of wafer‐scale photodetector array fabricated by using the proposed MoS_2_ separation‐transfer strategy and b) device configuration of the flexible MoS_2_ photodetector with graphene inserting layer serving as the photocarrier transportation channel. c) OM image of the flexible MoS_2_ photodetector with a channel length of 800 µm. The scale bar is 200 µm. d) Schematic diagram for photocarrier generation, separation, and transportation of photodetector based on the graphene‐MoS_2_ junction. The arrows point out the movement of photocarriers, and the dashed box represents the recombination of drifted electrons and pristine holes in graphene. e) Photocurrent comparison for flexible MoS_2_ photodetector with graphene channel and rigid photodetector based on bare MoS_2_ on sapphire. f) Schematic mapping of a 4 × 5 photodetector array for the letter “C” image, which is generated by an optical mask. g) Truth image of the electrical signal obtained by the photodetector array, and the color scale represents the current difference between illuminated and dark conditions. h) Schematic diagram of homemade bending test equipment. The flexible photodetector is appressed onto a copper foil and its curvature radius is controlled by the distance of two counter clamps. The original distance is D_0_ (flat), and that after bending is D_1_. i) Relative photocurrent variation of MoS_2_‐based flexible photodetector under the bending cycle test and j) bending angle test. The insets are photos of the real bending status for the flexible photodetector.

As the graphene inserting layer together with MoS_2_ is separated and transferred onto the resin, the graphene previously underneath the MoS_2_ now faces outside. As shown in **Figure** **6**a,b, the flexible photodetector is simply fabricated by depositing a pair of Au/Ti electrodes, which belongs to a typical metal‐semiconductor‐metal device configuration. The zoom‐in OM image of one as‐fabricated photodetector is shown in Figure 6c, the photosensitive area is defined by two counter electrodes as 800 × 800 µm^2^. According to the transmittance spectra in Figure 3b, the graphene layer absorbs less incident light for the wavelength above 450 nm. Hence, the MoS_2_ layer acts as the main photosensitive absorber, forming a heterojunction with the graphene, in which the latter provides a heterojunction electric field and serves as the channel for photocarrier separation and transportation, respectively.

The photoresponse ability of MoS_2_‐based flexible photodetector is evaluated by illuminating it with a 532 nm laser, in addition, the MoS_2_ sulfuration from MoO_x_ precursor on bare sapphire without annealing is used for fabricating the referenced device. The current‐voltage (I‐V) plots in dark and light conditions are shown in Figure S18 (Supporting Information), and the photocurrent is calculated by their difference in Figure 6e. Due to the high electrical conductivity and carrier mobility of graphene, the photocurrent of flexible photodetector is ≈10^5^ higher than the referenced device without a graphene channel. We attribute the largely enhanced responsivity to the high separation efficiency of the photocarrier induced by the junction electric field and high photocarrier collection efficiency from the graphene channel, as schematically depicted in Figure 6d. The current‐voltage‐time (I‐V‐T) plot of the flexible photodetector is shown in Figure S19 (Supporting Information), exhibiting the periodic response of the flexible photodetector to the frequency light input.

The responsivity (*R*) and specific detectivity (D^*^) of the photodetector could be calculated by the following equations:

(1) $$R=\frac{I_{\mathrm{light}}-I_{\mathrm{dark}}}{P}$$

(2) $$D^{*}=R\sqrt{\frac{A}{2qI_{\mathrm{dark}}}}$$

where *I_light_* and *I_dark_* are the currents measured under light and dark conditions, *P* is the effective light power, *A* is the detection area, and *q* is the elementary charge. The effective power of 532 nm laser is 19.8 mW, the *I_light_* and *I_dark_* are measured as 1.674 mA and 1.810 mA at the bias of 10 V, respectively. Therefore, the flexible photodetector obtains a responsivity of 6.86 mA W^−1^, and the specific detectivity is calculated as 2.28 × 10^7^ Jones.

According to the Raman spectra in Figure 2f and Figure 3a, the high‐temperature annealing and MoS_2_ growth have induced additional defects in the graphene lattice. In order to evaluate its effect on device degradation, the as‐grown MoS_2_ on graphene‐modified sapphire substrate is covered by a newly transferred graphene layer as the photocarrier transportation channel, the corresponding device has a higher photocurrent at the order of ≈10^−3^ A and responsivity of 145.3 mA W^−1^ (Figure S20, Supporting Information), proving a feasible route to further enhance the response of MoS_2_‐based flexible photodetector. In addition, the flexible photodetector based on the MoS_2_ grown by the vapor reaction of sulfur and MoO_x_ powders with higher crystalline quality was fabricated. As shown in Figure S21 (Supporting Information), the device reaches a high responsivity of 218.7 mA W^−1^. It reveals that the performance of flexible devices based on TMDCs by using this separation‐transfer strategy could be easily optimized by the graphene inserting layer and TMDCs material itself. As shown in **Table** **2**, it shows that our photodetectors enable excellent responsivity in contrast to similar MoS_2_‐based devices reported previously.

**Table 2:** Summaries of response characteristics, measurement conditions, and bending stability for MoS_2_‐based photodetectors.

MoS_2_ Photodetector	Wavelength	Power	Bias Voltage	Photosensitive Area	Responsivity	Bending Cycles @ Radius [Relative Responsivity]	Ref.
Flexible	240 nm	8 µW	5 V	1.2 × 10^5 ^µm^2^ (for one finger)	1.1 mA W^−1^	500 cycles @ 0.87 cm [≈100%]	[40]
Flexible	450 nm	98 pW	1 V	135.6 µm^2^	490.3 mA W^−1^	5000 cycles @ 1.5 cm [94.4%]	[41]
Flexible	660 nm	0.4 µW	1 V	≈1 × 10^4^ µm^2^	8.45 µA W^−1^	500 cycles @ 2.3 cm [≈82%]	[44]
Flexible	450 nm	49.32 nW	2 V	137 µm^2^	22.6 mA W^−1^	1 cycle @ 1.5 cm [≈83%]	[45]
Rigid	650 nm	1 nW	1 V	50 µm^2^	800 mA W^−1^	/	[46]
Rigid	445 nm	8 mW	10 V	1.2 × 10^5^ µm^2^	50.7 mA W^−1^	/	[47]
Rigid	650 nm	94 µW	0.1 V	≈1×10^8 ^µm^2^	32 mA W^−1^	/	[48]
Flexible	532 nm	19.8 mW	10 V	6.4 × 10^5^ µm^2^	6.86 mA W^−1^	5000 cycles @ 3.84 cm [≈100%]	This work
Rigid					145.3 mA W^−1^	/	

For imaging applications, a letter “C” image generated by an optical mask is illuminated on a 4×5 photodetector array, as schematically shown in Figure 6f. The truth image of the electrical signal in Figure 6g is calculated by the current difference between measured and initial conditions at the bias voltage of 10 V. The photodetector pixel shows large current variation as it is illuminated, which is 1–2 orders of magnitudes higher than the other pixels apart from the letter “C”. Therefore, the as‐fabricated photodetector array is featured with excellent imaging ability, ensuring the reproduction of the target image with high contrast and excellent accuracy.

The mechanical stability of the MoS_2_‐based flexible photodetector is measured by homemade bending test equipment, as shown in Figure 6h, and it could confine the device bending at diverse cycles and curvature radius. To make the bending test more controllable, the flexible photodetector is appressed onto a copper foil, and the counter clamps fasten the boundaries of the copper foil. The bending of copper foil together with a flexible photodetector is achieved by controlling the distance between two counter clamps. The bending curvature radius (*r*) of a flexible photodetector could be calculated by the original distance of *D*_0_ and *D*_1_ after bending following a relationship as:

(3) $$r=\frac{D_{0}}{\theta }$$

where *θ* is the angle corresponding to arc length (*D*_0_) in circles, which is solved by numerical calculation, as written in the formula below:

(4) $$\frac{\sin\frac{\theta }{2}}{\frac{\theta }{2}}=\frac{D_{1}}{D_{0}}$$

The photos of the controlled device bending status are exhibited in the inset of Figure 6i,j, for each acquired data point, it is calculated by nine photocurrent values from their I‐V‐T plots. The bending cycle test is first carried out at a constant curvature radius of ≈3.84 cm, and the flexible photodetector remains nearly 100% of its initial performance after 5000 bending cycles (Figure 6i). The bending stability of this MoS_2_‐based flexible photodetector is superior to the other works summarized in Table 2. Interestingly, we observe the starting enhancement and following reduction of photocurrent as the curvature radius gradually changes smaller (Figure 6j). The surface morphology of MoS_2_ on resin after bending of a small curvature radius (≈0.6 cm) is examined by SEM in Figure S22 (Supporting Information), the wrinkled structures are newly observed, and they were created by the compressive force since the bending direction is toward into the MoS_2_ surface. The wrinkles of MoS_2_ photosensitive absorber have a scattering effect on the illuminated light, and thus it would increase the light utilization efficiency and result in higher photocurrent.^[^ ^49^ ^]^ As a trade‐off, when the curvature radius decreases to the threshold value, the mechanical damage of the flexible photodetector takes the dominating effect and the device photocurrent shows slight degradation.

In contrast, for the flexible photodetector with thinner MoS_2_ grown by the vapor reaction of sulfur and MoO_x_ powders, as shown in Figure S23 (Supporting Information), it deteriorates rapidly to ≈60% of its initial photocurrent after 500 bending cycles. Even worse, the device goes into invalid after 1000 bending cycles, and no response can be observed. In contrast to the device with thicker MoS_2_ in Figure 6i, the bending stability of this flexible photodetector is relatively poor. The discussions about the bending stability reduction of the device are established in the Supporting Information. It reminds us that the design of flexible devices based on TMDCs should carefully consider the material thickness, thus balancing the response performance and bending stability.

## 3 Conclusion

In summary, the present work demonstrates a novel strategy for the growth and easy separation‐transfer of the waler‐scale MoS_2_ array via the growth interface design, in which the graphene inserting layer not only provides the weak van der Waals force but also promotes the high‐quality and uniform MoS_2_ growth. The HRTEM image exhibits excellent integrity of the graphene inserting layer after the annealing and sulfurization. The theoretical calculations prove that the graphene inserting layer efficiently screens the lattice field effect from the sapphire substrate, thus suppressing the chemical bonding formation between the MoS_2_ and sapphire. By patterning the MoS_2_ layer into an array, the easy separation and transfer enabled by a resin mediator achieves a high yield of ≈99.83%, and it also shows high applicability for the other TMDCs material grown with different methods. Meanwhile, the resin could also serve as a flexible substrate for device fabrication. Even better, the separable graphene layer together with MoS_2_ is valuable as a photocarrier transportation channel, resulting in ≈10^5^ enhancement for the photocurrent, and the corresponding responsivity of MoS_2_‐based flexible photodetector reaches 6.86 mA W^−1^. The 4×5 photodetector array is also capable of imaging, the truth image reproduced by electrical signal shows high contrast. Moreover, the flexible photodetector possesses excellent bending stability, it could remain at almost 100% of its initial performance after 5000 bending cycles, and work properly at the minimum bending curvature radius of 0.49 cm. The proposed strategy for TMDCs growth and processing meets the advantages of time‐saving, mass‐production, high material quality, and process controllability, which lightens the bright future for TMDCs in the next‐generation curved/wearable optoelectronic applications.

## 4 Experimental Section

### Graphene Synthesis and Transfer

The graphene was grown on the copper foil (Alfa Aesar) by a low‐pressure CVD (LPCVD) system (OTF‐1200X, HF‐Kejing) at 1000 °C with the gas of Ar, H_2_, and CH_4_ for 40 min. After that, the photoresist (S1805G) was spin‐coated onto the graphene/copper foil as a supporting layer. Then, the copper foil was etched away by Na_2_S_2_O_8_ solution, and the graphene supported by S1805G was dragged onto a sapphire substrate. Finally, the S1805G photoresist film was dissolved by 80 °C hot acetone.

### Annealing of MoO_x_ Precursor and MoS_2_ Growth

The MoO_x_ film of 2–7 nm was deposited on graphene/sapphire substrates and bare sapphire substrates by a homemade thermal vacuum evaporation system with a growth rate of 0.3 nm s^−1^. Then, the sample was covered with a SiO_2_ capping layer deposited by the plasma‐enhanced chemical vapor deposition (PECVD, Oxford Plasmalab System100). After that, the sample was annealed by a rapid heat annealing furnace at 800 °C for 30 min. Then, the sample was dipped into BOE (10:1) for 20 min to remove the SiO_2_ capping layer. The annealed MoO_x_ film was cleaned by deionized water 3 times. Finally, the annealed MoO_x_ sample was sulfurized in another double thermal zone LPCVD system (BTF‐1200C‐II‐L‐SL‐CVD). The 1 g sulfur powder was placed upstream and provided a S‐rich environment. The 150 sccm Ar carrier gas was applied and generated a pressure of 3–5 mbar. The samples were placed in the heating zone with a high temperature of 800 °C, and the sulfur powder was heated to 180 °C. After 1 h sulfurization, the system was naturally cooled to room temperature. The MoS_2_ grown by CVD with the sulfur and MoO_x_ powders was severed as the reference, it was carried out by LPCVD system (BTF‐1200C‐II‐L‐SL‐CVD) at 800 °C with the gas of Ar for 20 min, and 0.5 g sulfur and 0.05 g MoO_x_ powders were placed in the upstream as precursors. To achieve the patterned MoS_2_ array, a standard photo etching technique was carried out by using a photoresist (AZ5214E) as the anti‐etching layer, and the exposed region of MoS_2_ layer was etched by Ar plasma (RIE, SENTECH‐SI591).

### Annealing of WO_x_ Precursor and WS_2_ Growth

The WO_x_ film of 5 nm was also deposited on graphene/sapphire substrates by the homemade thermal vacuum evaporation system. After that, the SiO_2_ capping layer was deposited onto the sample by the plasma‐enhanced chemical vapor deposition (PECVD, Oxford Plasmalab System100), and then it was annealed at 800 °C for 30 min. After removing the SiO_2_ capping layer, it was sulfurized for the WS_2_ growth (BTF‐1200C‐II‐L‐SL‐CVD).

### Separation‐Transfer of MoS_2_ and Flexible Photodetector Fabrication

The UV photosensitive resin (NOA63) was spin‐coated onto the patterned MoS_2_ array as a mediator for the separation and transfer. After the UV exposure by a mercury lamp, the resin becomes solidification and converts into a flexible substrate. Then, the edge of the cured resin was carefully cut with the blade, and the MoS_2_ together with the graphene inserting layer was easily separated from the sapphire substrate by using tweezers to provide the mechanical force. The patterned Au/Ti (50/10 nm) electrodes were deposited on the graphene/MoS_2_/resin by thermal vacuum evaporation (QHV‐D86E), the photosensitive area was defined as 800 × 800 µm^2^.

### Characterization and Measurement

The surface morphology was measured by SEM (S‐4800, Hitachi), AFM (MultiMode8, Bruker) in tapping mode, OM (DSRi2, Nikon), and TEM (JEM‐2100F, JEOL). The sectional information was measured by TEM (TF20, FEI). The structural properties of MoO_x_ were evaluated by XRD (D8 Discover, Bruker). The materials properties of graphene, MoO_x_, and TMDCs were measured by Raman spectrometer (LabRAM HR Evolution, HORIBA Scientific) with the excitation laser of 532 nm and scanning spectrophotometer (UV‐3101 PC). The chemical component and bonding of MoS_2_ film was evaluated by XPS (EscaLab 250Xi, Thermo). The current‐voltage curve was collected by a KEITHLEY 2400 Source Meter and a PDA FS‐Pro 380 semiconductor analyzer, and a 532 nm laser was used as the light source. The periodic optical signal of the laser was controlled by an Arbitrary Function Generator (GW Instek AFG‐2225). For the imaging of 4×5 photodetector array, letter “C” image was generated through an optical mask. The bending test was carried out by a homemade system including a displacement platform, and the flexible photodetector was stuck on a piece of copper foil. Hence, the copper foil bending exhibited the flexuosity of the flexible photodetector.

### Theoretical Calculations

All the theoretical calculations were based on density functional theory (DFT) using the Vienna ab initio simulation package.^[^ ^50^ ^]^ The Perdew‐Burke‐Ernzerhof was used for the exchange‐correlation potential of valence electrons, and the projector‐augmented wave method was employed for the core region.^[^ ^51^, ^52^ ^]^ The cutoff energy was set to 400 eV. The van der Waals interaction was described by DFT‐D3 correction.^[^ ^53^ ^]^ The structures of MoS_2_/graphene/sapphire and MoS_2_/sapphire were investigated for the binding energy. A 2 × 2 × 1 supercell for sapphire, a 4 × 4 × 1 supercell for graphene, and a 3 × 3 × 1 supercell for MoS_2_ were used to minimize the in‐plane lattice mismatch (<3%) and to avoid the imaginary interaction between the defects. The vacuum layer was set to be larger than 20 Å. The binding energy was defined by *E_binding_ = E_tot_ – E_sub_ – E_film_*, where *E_tot_*, *E_sub_*, and *E_film_* represent the free energies of the complexes, the sapphire substrate, and the grown film on the sapphire, respectively. The negative values represent an exothermic process. During all structural relaxation, the bottom 2 atomic layers of sapphire were fixed. The *k*‐mesh of 0.04 and 0.03 2 π Å^−1^ was used for atomic relaxations and self‐consistent calculations, respectively. The convergence criteria for energy and force were chosen as 10^−4^ eV and 0.01 eV Å^−1^, respectively.

## Conflict of Interest

The authors declare no conflict of interest.

## Supporting information

Supporting Information

## Data Availability

Research data are not shared.
