# Supplementary material for: Wafer‐Scale Growth and Transfer of High‐Quality MoS2 Array by Interface Design for High‐Stability Flexible Photosensitive Device
Source: Adv Sci (Weinh). 2024 Jul 7;11(34):2405050. doi: 10.1002/advs.202405050 (PMC11425836; doi:10.1002/advs.202405050)
Supplement: Supplementary file 1 — Supporting Information [file ADVS-11-2405050-s001.pdf]

ADVANCED SCIENCE
Open Access

Supporting Information

for *Adv. Sci.*, DOI 10.1002/advs.202405050

Wafer-Scale Growth and Transfer of High-Quality MoS_2 Array by Interface Design for High-Stability Flexible Photosensitive Device

Bingchen Lü, Yang Chen, Xiaobao Ma, Zhiming Shi, Shanli Zhang, Yuping Jia, Yahui Li, Yuang Cheng, Ke Jiang, Wenwen Li, Wei Zhang, Yuanyuan Yue, Shaojuan Li, Xiaojuan Sun* and Dabing Li**

Supporting Information

Wafer-Scale Growth and Transfer of High-Quality MoS_2 Array by Interface Design for High-Stability Flexible Photosensitive Device

Bingchen Lü, Yang Chen, Xiaobao Ma, Zhiming Shi, Shanli Zhang, Yuping Jia, Yahui Li, Yuang Cheng, Ke Jiang, Wenwen Li, Wei Zhang, Yuanyuan Yue, Shaojuan Li, Xiaojuan Sun,* Dabing Li**

B. Lü, Y. Chen, X. Ma, Z. Shi, S. Zhang, Y. Jia, Y. Li, Y. Cheng, K. Jiang, S. Li, X. Sun, D. Li

Key Laboratory of Luminescence Science and Technology, Chinese Academy of Sciences & State Key Laboratory of Luminescence and Applications

Changchun Institute of Optics, Fine Mechanics and Physics

Chinese Academy of Sciences

Changchun 130033, P. R. China

E-mail: cheny@ciomp.ac.cn

E-mail: sunxj@ciomp.ac.cn

E-mail: lidb@ciomp.ac.cn

B. Lü, Y. Chen, X. Ma, Z. Shi, S. Zhang, Y. Jia, Y. Li, Y. Cheng, K. Jiang, S. Li, X. Sun, D. Li

Center of Materials Science and Optoelectronics Engineering

University of Chinese Academy of Sciences

Beijing 100049, P. R. China

W. Li, W. Zhang

Key Laboratory of Automobile Materials MOE, and School of Materials Science & Engineering, and Electron Microscopy Center, and International Center of Future Science, and Jilin Provincial International Cooperation Key Laboratory of High-Efficiency Clean Energy Materials

Jilin University

Changchun 130012, P. R. China

Y. Yue

School of Management Science and Information Engineering

Jilin University of Finance and Economics

Changchun 130117, P. R. China

1. Surface morphology comparison for different capping layers

The MoO_x/graphene/sapphire samples are covered by three kinds of capping layers, 50 nm SiO_2, 100 nm SiO_2, and 50 nm Si_3N_4, by the plasma enhanced chemical vapor deposition (PECVD) system, respectively, all samples are annealed at 800 °C for 30 min. As shown in Figure S1, the optical microscope (OM) images exhibit significant differences among these three samples. The capping layer of 50 nm SiO_2 (Figure S1a) maintains stability after the annealing. However, the capping layer of 100 nm SiO_2 (Figure S1b) appears numerous bubbles. One possible reason for the bubbles is that the gaseous products were generated from the MoO_x and cannot directly

overflow through a thick SiO_2 100 nm capping layer, while for the thinner 50 nm capping layer, it less prevents the gas escaping. The capping layer of 50 nm Si_3N_4 shows serious crack and self-exfoliation (Figure S1c). Based on the thermal expansion coefficients of SiO_2 (~0.6×10^{-6} K^{-1}), Si_3N_4 (~2.44×10^{-6} K^{-1}) and MoO_x (~0.6×10^{-6} K^{-1}), the difference of thermal expansion coefficients between Si_3N_4 and MoO_x is larger than that of SiO_2.[1-3] Hence, it is reasonable that the Si_3N_4 capping layer seriously cracks due to the large stress generation, which comes from the thermal expansion coefficient mismatch during the cooling process.

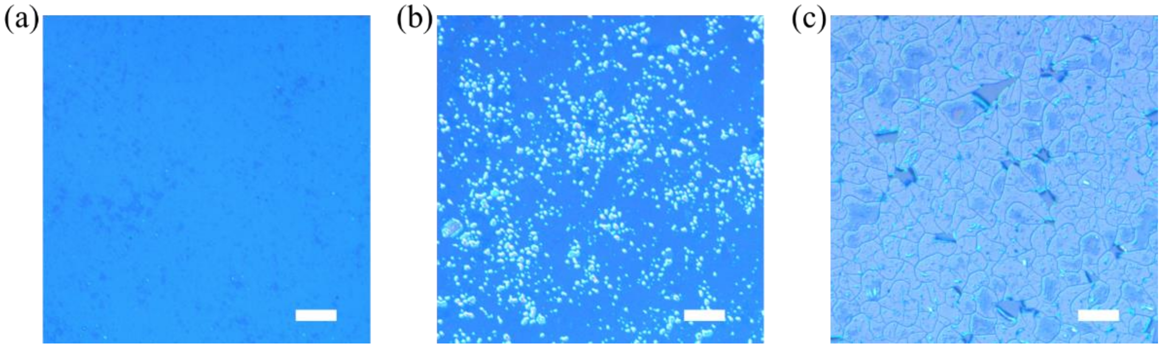

Figure S1. a) OM images of different capping layers of 50 nm SiO_2, b) 100 nm SiO_2 and c) 50 nm Si_3N_4 after 800 °C annealing for 30 min. The scale bar is 50 μm.

2. Surface morphology characterization of 50 nm SiO_2 capping layer after annealing

The atomic force microscope (AFM) is applied to investigate the surface morphology of 50 nm annealed SiO_2 capping layer in Figure S2. The height profile shows that the maximum particle height is ~16 nm, which is much smaller than the SiO_2 thickness of 50 nm. Hence, after the high-temperature annealing, the SiO_2 particles only form at its surface, and the SiO_2 capping layer remains continuous under these particles, and still providing excellent protection function.

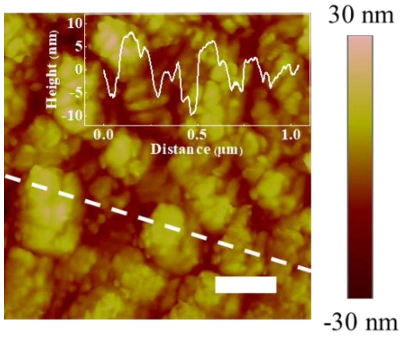

Figure S2. AFM image of 50 nm SiO_2 capping layer on the MoO_x precursor film after 800 °C annealing. The height profile along the dashed line is shown in the inset. The scale bar is 200 nm.

3. Surface morphology characterization of annealed MoO_x without SiO_2 capping layer

The MoO_x precursor is deposited on a bare sapphire substrate by vacuum thermal evaporation. After it was annealed without a SiO_2 capping layer, the AFM image of MoO_x precursor is featured with discrete clusters, whose size is larger than 80 nm in diameter, as shown in Figure S3. Without a SiO_2 capping layer, the annealing process has greatly broken the continuity of MoO_x film.

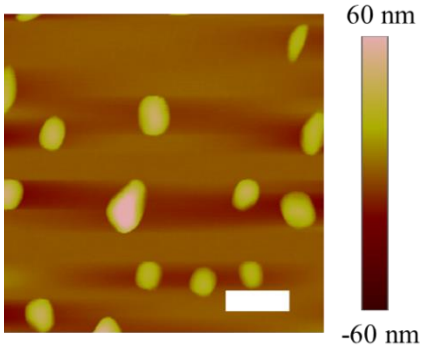

Figure S3. AFM image of the MoO_x precursor on bare sapphire without SiO_2 capping layer after the high-temperature annealing. The scale bar is 200 nm.

4. Raman measurement for MoO_x

The enlarged Raman spectra of MoO_x on graphene/sapphire (Figure 2f of the main manuscript) before and after the annealing, the typical Raman bands of MoO_x only exhibit for the annealed sample, as shown in Figure S4. Hence, the crystalline quality of MoO_x precursor is assuredly enhanced by the annealing treatment.

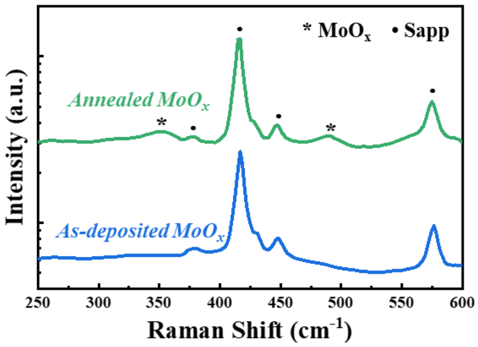

Figure S4. Enlarged Raman spectra from Figure 2f (main manuscript) for the annealed and as-deposited MoO_x on the graphene-modified sapphire substrate.

5. Stability evaluation of annealed MoO_x to BOE treatment

Two samples of 5 nm MoO_x deposited on graphene-modified sapphire substrate with the SiO_2 capping layer are used to illustrate the annealing effect for the MoO_x stability enhancement. After being disposed by Buffered Oxide Etch (BOE), the annealed MoO_x shows dark black color, but the unannealed sample becomes semitransparent (Figure S5a). The Raman spectra of unannealed sample after sulfuration shows none MoS_2-related bands (Figure S5b), indicating that the as-deposited MoO_x is resistless to the BOE solution and removed from the graphene inserting layer during this treatment. As for the annealed MoO_x, the excellent chemical stability

results in the successful growth of MoS_2.

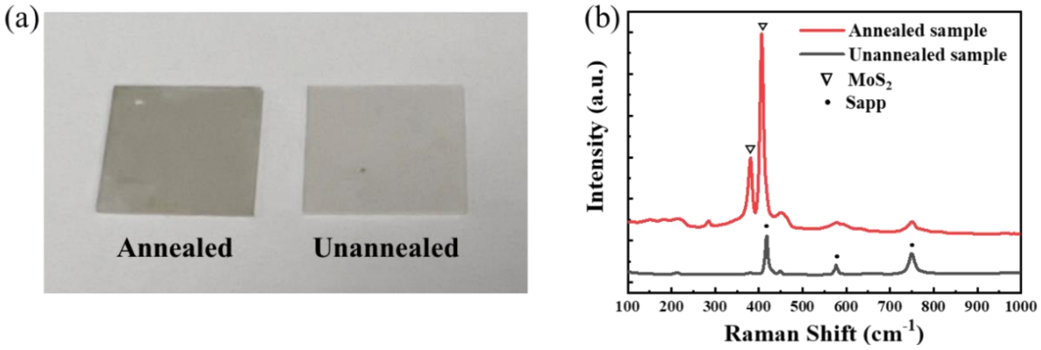

Figure S5. a) Photos of annealed and unannealed MoO_x on graphene-modified sapphire after treating by BOE solution. b) Raman spectra of samples in (a) after sulfuration.

6. Raman measurement of MoS_2 film with different thickness

The Raman spectra of MoS_2 grown from MoO_x precursor with different thicknesses (from 2 nm to 7 nm) exhibit gradual intensity variation and a series difference of E_{2g} and A_{1g} band position, as shown in Figure S6. Among these Raman spectra, the MoS_2 sample from thinner MoO_x precursor film has a weaker peak intensity and smaller peak position difference (A_{1g}-E_{2g}). The present MoS_2 growth strategy satisfies the high elasticity for MoS_2 thickness control.

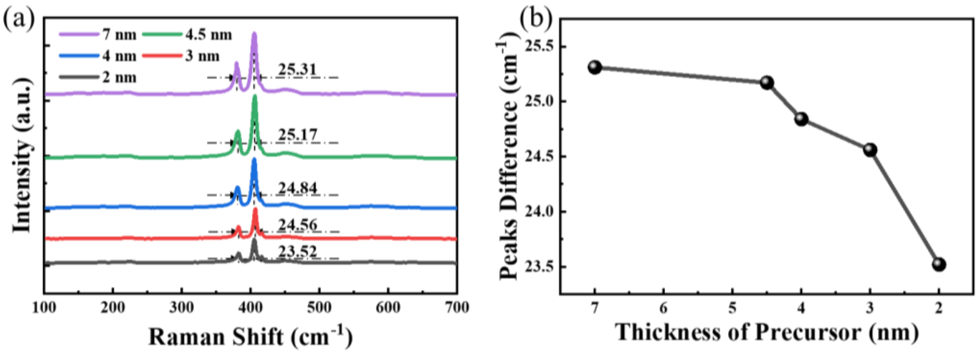

Figure S6. a) Raman spectra of MoS_2 films obtained by controlling the thickness of as-

deposited MoO_x precursor (2 nm, 3 nm, 4 nm, 4.5 nm, and 7 nm). b) Trend curve for the peak position difference of MoS_2 E_{2g} and A_{1g} band, extracting from (a).

7. Chemical components measurement of MoS_2

The X-ray photoelectron spectroscopy (XPS) survey scan for the MoS_2 grown from annealed MoO_x precursor with and without a graphene inserting layer is shown in Figure S7. The Mo 3d and S 2p peaks of MoS_2 are given in the survey scan.

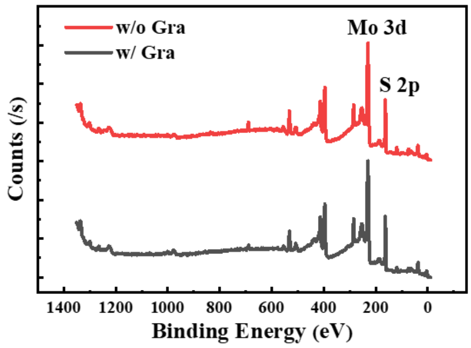

Figure S7. XPS survey scan of MoS_2 grown from annealed MoO_x with and without a graphene inserting layer.

8. EDX mapping measurement of MoS_2

In order to improve the electrical conductivity of samples during the Energy Dispersive X-ray spectroscopy (EDX) measurement, W metal is sprayed on their surface. The gaps between W and Al_2O_3 are the distribution of MoS_2/Graphene or MoS_2, which is consistent with the EDX mapping of Mo and S elements in Figure 3j and 3l of the main manuscript. On the other hand,

the top surface of MoS_2 grown without a graphene inserting layer in its cross-sectional transmission electron microscopy (TEM) image (Figure S8c) is rough, corresponding to the slit structures observed in its AFM image (Figure 3e of the main manuscript).

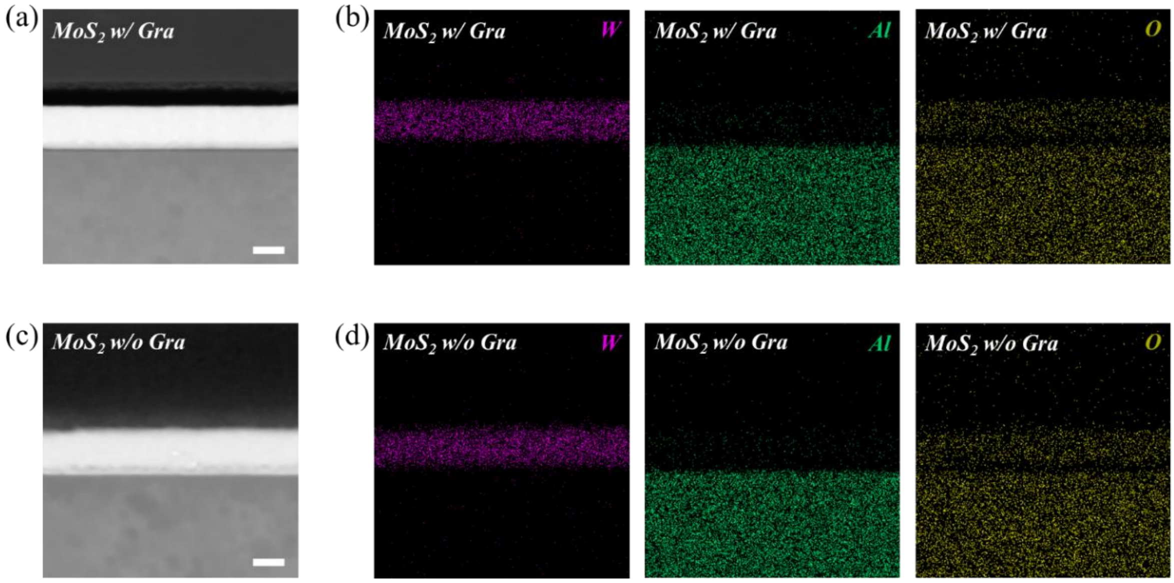

Figure S8. a) Low-magnification cross-sectional TEM images and b) EDX mapping of MoS_2 with a W metal spraying layer on the MoS_2 grown with and c) d) without a graphene inserting layer. EDX mapping shows the distribution of W, Al, and O elements. The scale bar in TEM images is 20 nm.

9. Element analysis and survey of MoS_2

The atom ratio of Mo, S, Si, and W elements was obtained from the EDX mapping in Figure S8, no Si element is presented on the MoS_2 film, no matter if it is grown with and without the graphene inserting layer. It proved that the SiO_2 capping layer could be completely etched by BOE solution, and no Si-related pollution on the as-grown MoS_2 film.

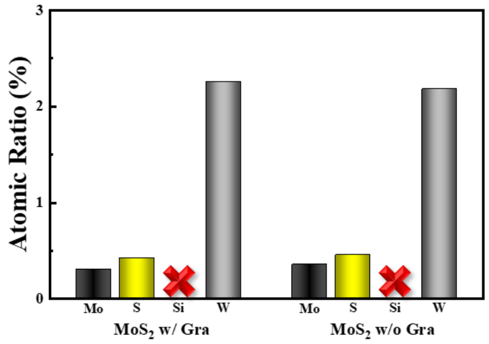

Figure S9. The summary of atom ratio of Mo, S, Si, and W elements for the MoS_2 films after removing the SiO_2 capping layer, which is obtained by the EDX measurement in Figure S8.

10. TEM characterization of MoS_2 grown by sulfurization of annealed MoO_x film

The MoS_2 film grown with the graphene inserting layer was mechanically separated from the sapphire substrate and suffered from an ultrasonic treatment in ethanol solution, which was then dripped onto a copper screen for the TEM test. The TEM image in Figure S10a shows the crystal domains and an apparent crystal lattice could be observed in these MoS_2 domains, as circled by different colored lines. Since the measured samples were prepared by the dropping method from the solution, the domain size and orientation of MoS_2 are different. The selected area electron diffraction (SAED) patterns in Figure S10b exhibit a relatively complex spot distribution, while it still tends to arrange in the hexagonal symmetry, according with the crystal structure of MoS_2.

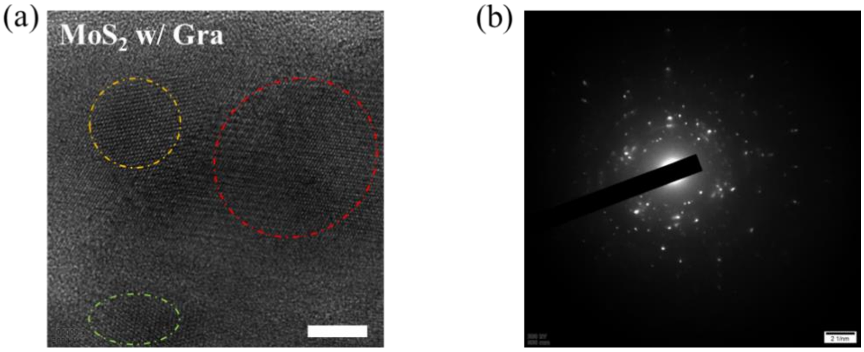

Figure S10. a) High-resolution TEM image and b) SAED pattern of MoS_2 grown with the graphene inserting layer. The scale bar is 5 nm.

11. TEM characterization of MoS_2 grown by vapor reaction of sulfur and MoO_x powers

The MoS_2 grown by vapor reaction of sulfur and MoO_x powers (CVD) was also grown and evaluated by the TEM and SAED measurements, the measured sample was prepared and exactly identical to that in Figure S10. This MoS_2 also shows a typical lattice structure in its high-resolution TEM image (Figure S11a). The diffraction spot array with hexagonal symmetry of the SAED pattern in Figure S11b is clearer than that in Figure S10, demonstrating the higher crystal quality of MoS_2 material grown by CVD.

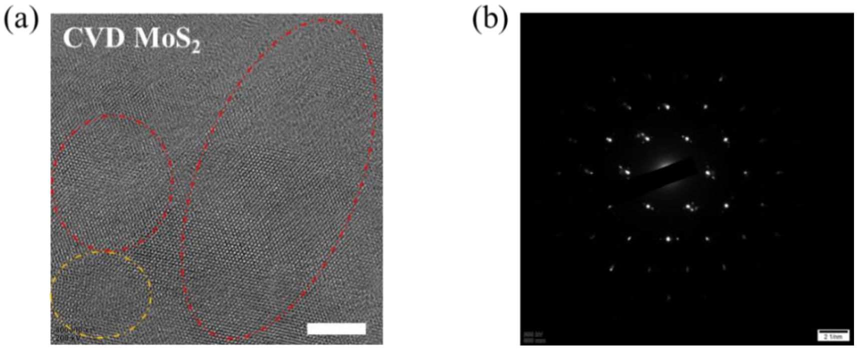

Figure S11. a) High-resolution TEM image and b) SAED pattern of MoS_2 grown by CVD. The scale bar is 5 nm.

12. Confirmation for the reliability of this separation-transfer strategy

As shown in Figure S12, with half graphene coverage and the rest exposure on the same sapphire substrate, the sample directly proves the reliability of our proposed MoS_2 growth and transfer strategy. It obviously shows that only the MoS_2 grown on the graphene inserting layer could be intactly separated by simply applying a mechanical force with an ultraviolet (UV) resin.

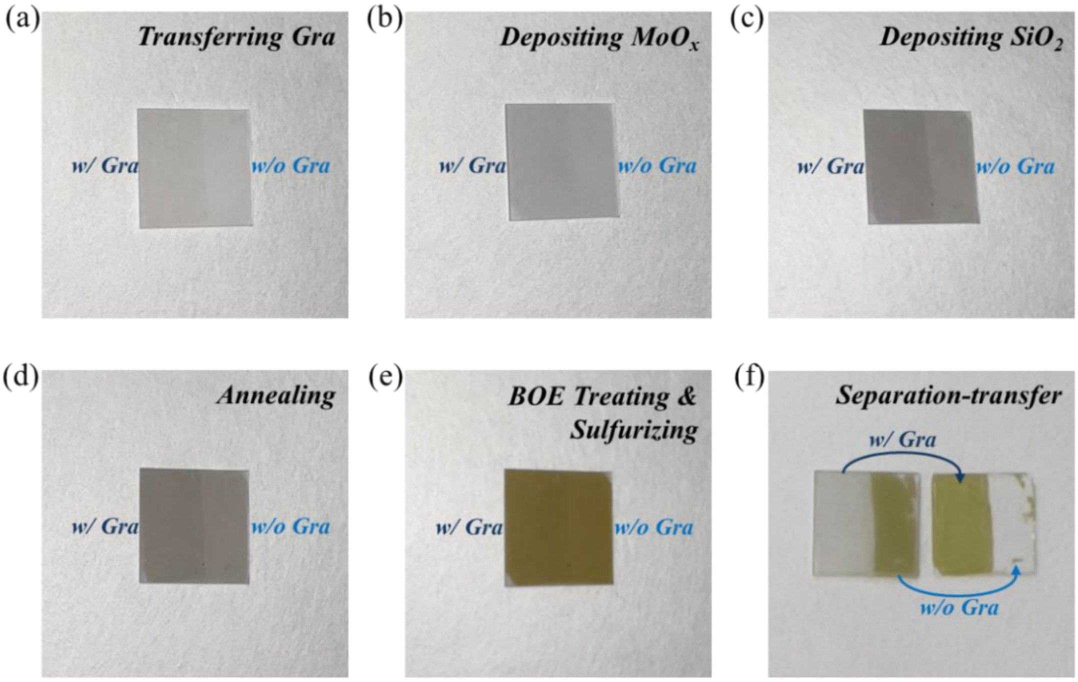

Figure S12. Photos for the compared separation-transfer of MoS_2 film with and without a graphene inserting layer. a) After transferring a graphene layer on the half of sapphire substrate. b) After deposition of MoO_x precursor on the substrate by vacuum thermal evaporation. c) After deposition of SiO_2 capping layer on the MoO_x by PECVD. d) After annealing in N_2 atmosphere. e) After etching the SiO_2 capping layer by treating it with BOE and sulfurizing the sample. f) After mechanical separation-transfer of MoS_2 by using UV resin. Only the left part of the MoS_2 film with the graphene inserting layer could be intactly separated and transferred.

13. Separation-transfer strategy applied for the MoS_2 grown by CVD

The MoS_2 grown by CVD, with a half graphene layer coverage on the sapphire substrate, is mechanically separated by using a UV resin. Without the graphene inserting layer, most of the MoS_2 layer grown on these regions remains on the sapphire substrate, which could not be intactly separated and transferred onto the UV resin, corresponding to the higher interfacial adhesion. As a comparison, the MoS_2 grown on graphene inserting layer by CVD has been fully separated and transferred, further revealing the applicability of this separation-transfer strategy.

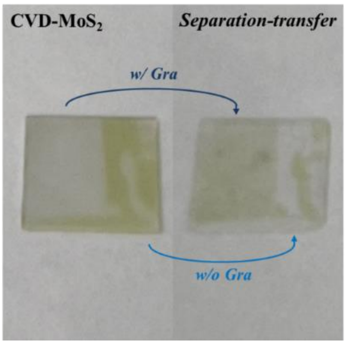

Figure S13. Photography of CVD-grown MoS_2 on the sapphire substrate covered with a half of graphene inserting layer (left) and UV resin (right) after the mechanical separation-transfer. The left part of MoS_2 grown on the graphene inserting layer could be intactly separated.

14. Morphology comparison of patterned MoS_2 unit over the separation-transfer

The scanning electron microscope (SEM) images of one MoS_2 unit in the patterned array before (on graphene/sapphire) and after separation-transfer (on resin) are compared in Figure S14. The clear edge and intact geometry of the patterned MoS_2 circle demonstrate the high reliability of the proposed separation-transfer strategy.

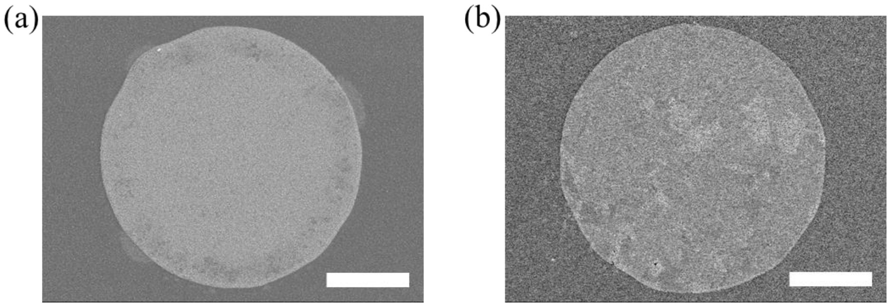

Figure S14. a) SEM image of a single MoS_2 unit from the patterned array before and b) after separation-transfer. The scale bar is 5 μm.

15. Summary of the separation-transfer yield for patterned MoS_2 array

To assess the MoS_2 separation-transfer efficiency and final yield, six different regions from the 2-inch wafer of patterned MoS_2 array on the resin are measured by OM and summarized, as shown in Figure S15 and Table S1. The separation-transfer of the patterned MoS_2 array achieves an extremely high yield of ~99.83%.

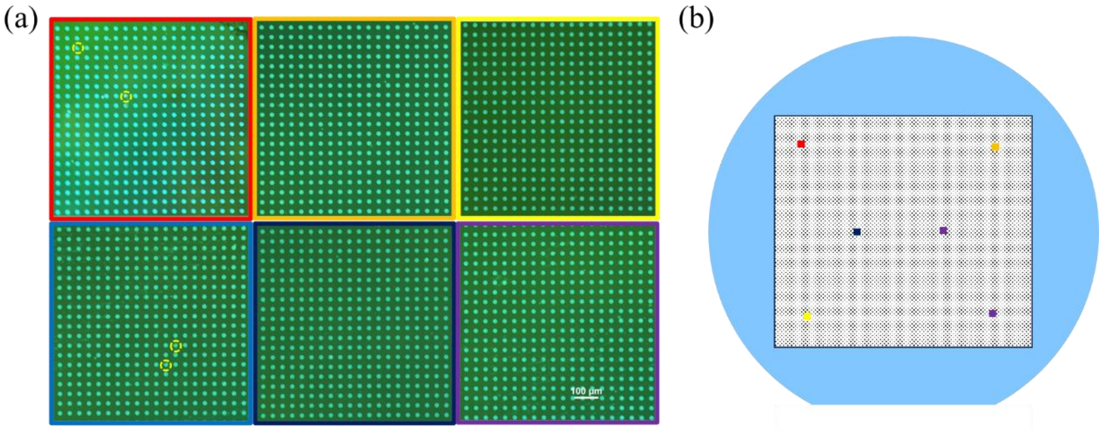

Figure S15. a) OM images of six different regions for the patterned MoS_2 array on the resin. b) Schematic diagram of six regions located on the 2-inch wafer.

Table S1. The yield of the separation-transfer of MoS_2 pattern arrays on resin.

Serial Number	MoS_2 Count	Failure Count	Failure Rate	Yield
1	400	2	0.5%	99.5%
2	400	0	0%	100%
3	400	0	0%	100%
4	400	2	0.5%	99.5%
5	400	0	0%	100%
6	400	0	0%	100%
Summary	2400	4	~0.17%	~99.83%

16. Applicability investigation of TMDCs grown on graphene inserting layer

Three kinds of transition metal disulfide compounds (TMDCs), including MoS_2 grown by CVD, MoS_2 grown by sulfurizing 1.5 nm MoO_x film, and WS_2 grown by sulfurizing 5 nm WO_x film on the graphene inserting layer, are prepared to evaluate the applicability of this separation-transfer strategy. These TMDCs materials were patterned into the circle array for the following test convenience. As shown in Figure S16a, the Raman spectra of these samples on the sapphire substrate exhibit typical bands of TMDCs materials (MoS_2 and WS_2) and the graphene inserting layer. For the MoS_2 films grown here, the difference between E_{2g} band and A_{1g} band is 22.77 cm^{-1} (MoS_2 grown by CVD) and 23.45 cm^{-1} (sulfurization from 1.5 nm MoO_x film), which are two layers and three layers MoS_2, respectively. Then, the height of the patterned TMDCs array

is measured by AFM, as shown in Figure S16b. The thickness of these TMDCs materials is slightly higher than their estimated layer number in Raman spectra of Figure S16a, which might be attributed to the accumulation of underneath graphene inserting layer.

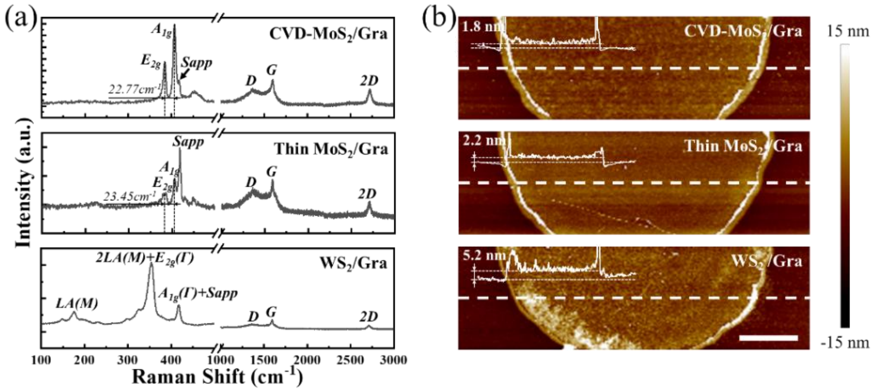

Figure S16. a) Raman spectra of three kinds of TMDCs, including MoS_2 grown by CVD, MoS_2 grown by sulfurizing 1.5 nm MoO_x film, and WS_2 grown by sulfurizing 5 nm WO_x film on the graphene inserting layer. b) Corresponding AFM images of these patterned TMDCs array. The insets show the height profiles of TMDCs patterns, the measured positions are marked by the white dash line. The scale bar is 5 μm.

17. Applicability investigation of this separation-transfer strategy for different TMDCs

As shown in Figure S17, the patterned TMDCs array in Figure S16 was separated and transferred onto the UV resin by using the proposed separation-transfer strategy. The separation-transfer efficiency for the MoS_2 grown by CVD and thick WS_2 grown by sulfurizing 5 nm WO_x film reaches a high yield of over 99.38%. However, for the relatively thin MoS_2 (sulfurization from 1.5 nm MoO_x film), it shows a relatively lower yield of ~97.22%. This might

be attributed to the higher defect density of graphene inserting layer after the high-temperature sulfurization, which is revealed by the enhanced defect-related D band intensity in its Raman spectra (Figure S16a).

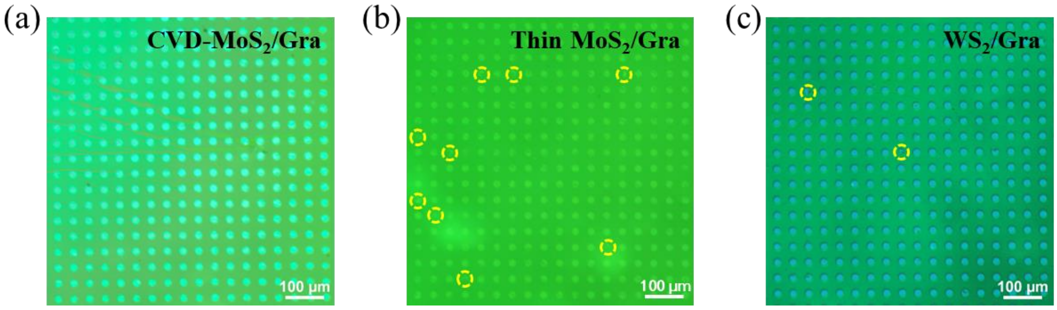

Figure S17. a) OM images of patterned MoS_2 array grown by CVD, b) MoS_2 array grown by sulfurizing 1.5 nm MoO_x film, and c) WS_2 array grown by sulfurizing 5 nm WO_x film after separating and transferring onto the UV resin.

18. I-V characteristics of MoS_2-based photodetector

The current-voltage (I-V) plots of the MoS_2-based flexible photodetector with a graphene photocarrier transportation channel and rigid device without a graphene channel are measured in Figure S18a and 18b, and the dark and light conditions are separately measured. The photocurrent in Figure 6e (main manuscript) is calculated by the difference between the measured current in dark and light conditions.

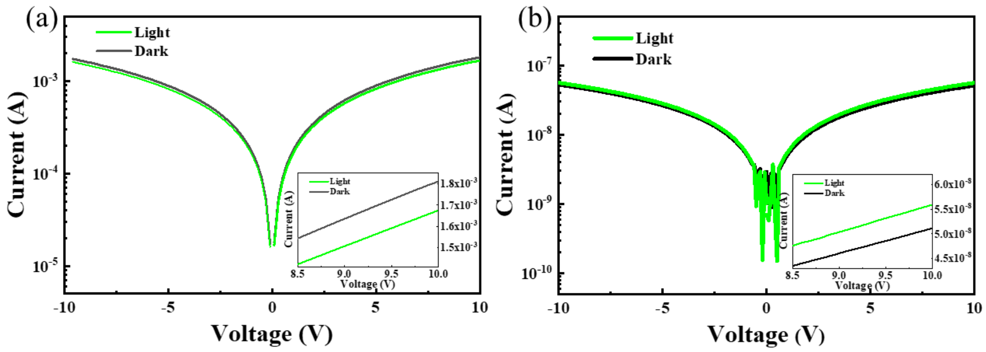

Figure S18. a) I-V plots of MoS_2-based flexible photodetector with graphene inserting layer as the photocarrier transportation channel and b) rigid photodetector based on the general MoS_2 grown on bare sapphire substrate in light and dark conditions.

19. I-V-T characteristics of MoS_2-based flexible photodetector

The MoS_2-based flexible photodetector is illuminated under a 532 nm laser controlled by a periodic signal. The current-voltage-time (I-V-T) plot of the flexible photodetector shows the periodic response to the frequency light input.

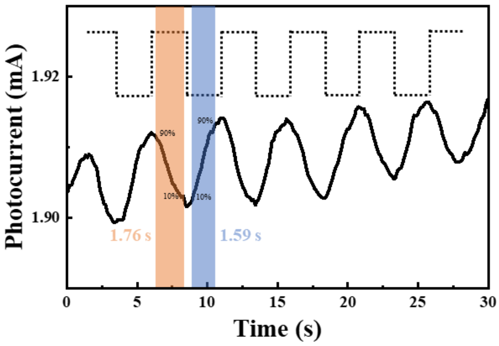

Figure S19. Response of MoS_2-based flexible photodetector to a periodic 532 nm laser illumination. The dashed line exhibits the modulated signal of a periodic optical pulse. The orange and blue blocks represent rise time and fall time.

20. Enhanced response performance of MoS_2-based photodetector by transferring a high-quality graphene as carrier transportation channel

Another graphene monolayer is transferred onto the as-grown MoS_2 on a graphene-modified sapphire substrate, serving as the photocarrier transportation channel. Then, Au/Ti electrodes are deposited to fabricate a photodetector. The I-V curves plotted in dark and light conditions and calculated photocurrent are shown in Figure S20, which has a higher photocurrent (at the order of ~10^{-3} A) and responsivity (145.3 mA W^{-1}) as the comparison with the MoS_2-based flexible photodetector with graphene inserting layer as the channel.

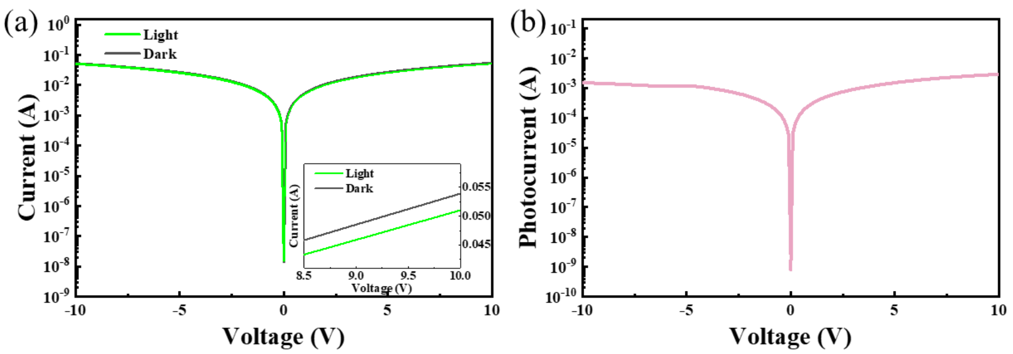

Figure S20. a) I-V plots in dark and light conditions, b) calculated photocurrent by the difference of measured current in (a) for the photodetector based on MoS_2 grown on sapphire with another transferred graphene layer as photocarrier transportation channel.

21. I-V and I-V-T characteristics of other photodetectors based on the MoS_2 with thinner thickness and higher crystalline quality

The flexible metal-semiconductor-metal (MSM) photodetectors based on the relatively thin MoS_2 grown by sulfurizing from 1.5 nm MoO_x film and CVD were prepared. The I-V curves

tested in dark and light conditions and calculated photocurrent curves are shown in Figure S21. The responsivity of flexible photodetector based on MoS_2 grown by sulfurizing from 1.5 nm MoO_x film possesses a 1.77 times enhancement (12.14 mA W^{-1}) in contrast to the thick MoS_2 in Figure 6e of the main manuscript. Here, the thinner 2D TMDCs exhibit a larger electro-optic coefficient (r^*_{33}) and greater refractive index, which is sensitive to the electric field, indicating a stronger electro-optic response.[4] Therefore, the flexible photodetector based on the thinner MoS_2 (sulfurization from 1.5 nm MoO_x film) obtains an enhanced responsivity. In addition, the flexible photodetector based on the MoS_2 grown by CVD has a much higher responsivity of 218.7 mA W^{-1}, benefiting from its relatively high crystal quality (Figure S11).

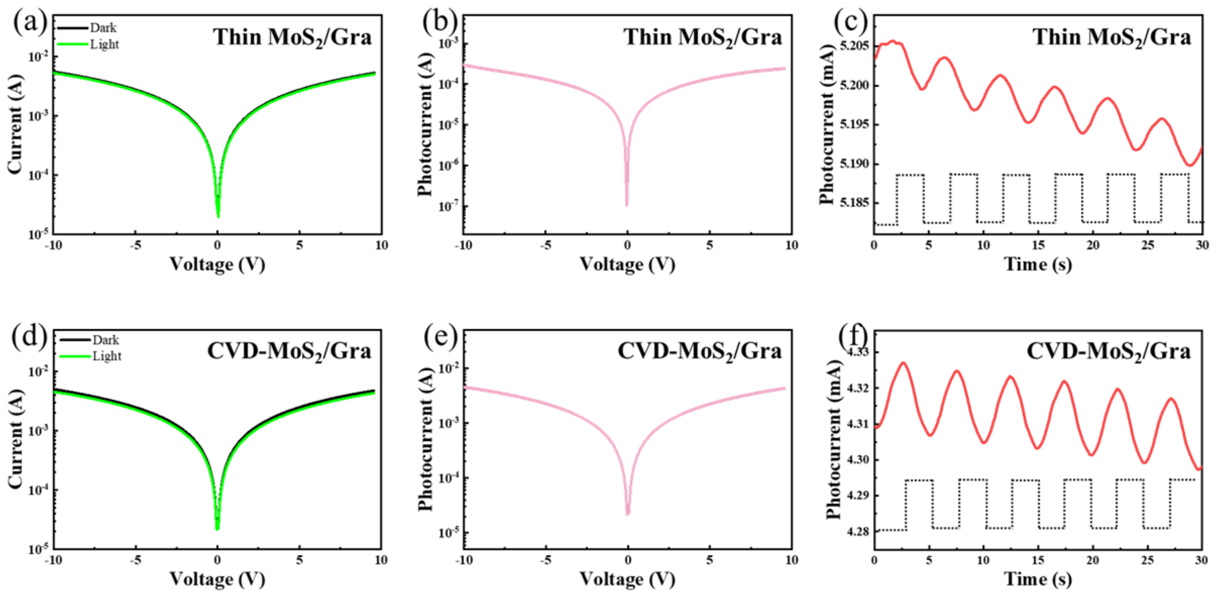

Figure S21. a) I-V plots of the flexible photodetectors based on the MoS_2 grown by sulfurizing from 1.5 nm and d) the MoS_2 grown by CVD under dark and light conditions. b) and e) The corresponding calculated photocurrent plots from that measured in (a) and (d). c) and f) The response of these two flexible photodetectors to a periodic 532 nm laser.

22. Morphology characteristic of the MoS_2 layer after bending

As shown in Figure S14, the surface of MoS_2 on resin after bending at a small curvature radius (~0.6 cm) exhibits wrinkled structures, which are newly observed.

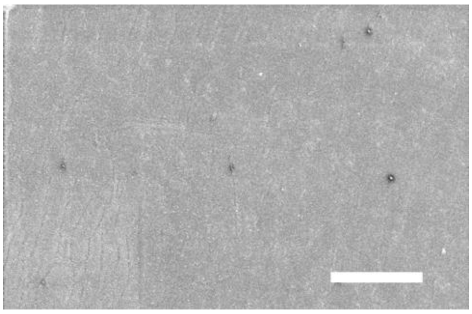

Figure S22. SEM image of MoS_2 on resin after bending at a small curvature radius. The scale bar is 5 μm.

23. Bending test of the MoS_2-based flexible photodetector grown by CVD

The bending cycle test of flexible photodetector based on the MoS_2 grown by CVD was carried out in Figure S22. It shows rapid deterioration after bending for 500 cycles, only maintaining ~60% of its initial photocurrent. Furthermore, after 1000 bending cycles, this device goes into invalid, and none response could be observed (Figure S22a). In contrast to the device based on thicker MoS_2 in Figure 3i of the main manuscript, the bending stability of the flexible photodetector here is really poor. During the bending test, cracks might be induced in the MoS_2 film, while the material with more layer numbers could provide increased conductive paths for photocarrier transportation, resisting the local cracks. Meanwhile, slip or deformation of MoS_2 is another reason for the performance degradation during device bending.[5] The MoS_2 grown

by sulfurizing from MoO_x film possesses numerous domain boundaries (Figure 2d of the main manuscript), which are severed as the releasing pathway for bending stress and benefit to high bending stability.

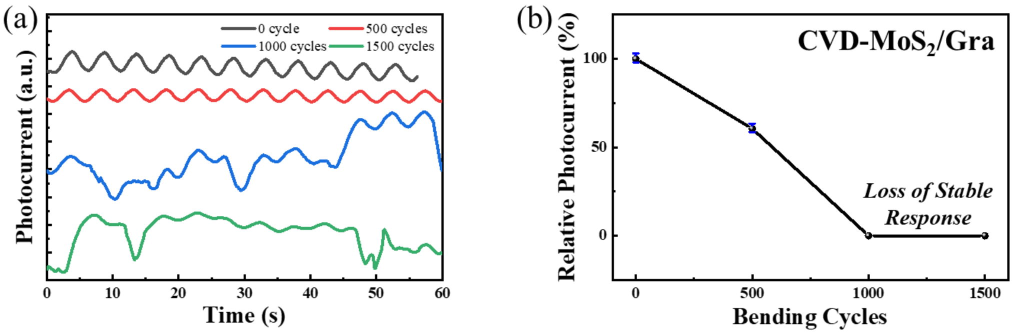

Figure S23. a) The response of the flexible photodetector to periodic light illumination at different bending cycles. b) Relative photocurrent dependent on the bending cycle.

References

[1] H. Tada, A. E. Kumpel, R. E. Lathrop, J. B. Slanina, P. Nieva, P. Zavracky, I. N. Miaoulis, P. Y. Wong, *J. Appl. Phys.* **2000**, *87*, 4189.

[2] A. K. Sinha, H. J. Levinstein, T. E. Smith, *J. Appl. Phys.* **2008**, *49*, 2423.

[3] H. Negishi, S. Negishi, Y. Kuroiwa, N. Sato, S. Aoyagi, *Phys. Rev. B* **2004**, *69*, 064111.

[4] A. Paul, I. Grinberg, *Phys. Rev. Appl.* **2022**, *17*, 024042.

[5] G. Wang, Z. Dai, J. Xiao, S. Feng, C. Weng, L. Liu, Z. Xu, R. Huang, Z. Zhang, *Phys. Rev. Lett.* **2019**, *123*, 116101.
